# The Impact of a Pulsed Light Stream on the Quality and Durability of the Cold-Stored Longissimus Dorsal Muscle of Pigs

**DOI:** 10.3390/ijerph20054063

**Published:** 2023-02-24

**Authors:** Paulina Duma-Kocan, Mariusz Rudy, Marian Gil, Renata Stanisławczyk, Jagoda Żurek, Grzegorz Zaguła

**Affiliations:** 1 Department of Agricultural Processing and Commodity Science, Institute of Food and Nutrition Technology, College of Natural Sciences, University of Rzeszow, Zelwerowicza 4, 35-601 Rzeszow, Poland; 2 Department of Financial Markets and Public Finance, Institute of Economics and Finance, College of Social Sciences, University of Rzeszow, Cwiklinskiej 2, 35-601 Rzeszow, Poland; 3 Department of Bioenergetics, Food Analysis and Microbiology, Institute of Food and Nutrition Technology, College of Natural Science, University of Rzeszow, Cwiklińskiej 2D, 35-601 Rzeszow, Poland

**Keywords:** meat quality, pulsed light, food security, physicochemical and technological properties, food safety

## Abstract

The purpose of this study was to investigate the effect of pulsed light application (exposure to a pulsed light beam (PL) of 400 Hz for a period of 60 s, with an energy dose of 600 mW and wavelengths of 660 and 405 nm) on the physicochemical, technological, and sensory properties, as well as the nutritional value and shelf life of cold-storage pig longissimus dorsi muscle. Each muscle was divided into six parts, three of which were control samples, and the rest were exposed to pulsed light. The detailed laboratory tests of the meat were conducted 1, 7, and 10 days after slaughter. The meat was cold stored at +3 °C ± 0.5 °C. The study showed that the application of pulsed light has a favorable effect on lowering the TBARS index, oxidation-reduction potential, and water activity values. In addition, the application of PL had no statistically significant effect on the variation in the perception of selected sensory characteristics of meat. Furthermore, PL processing, as a low-energy-intensive method that can be environmentally friendly and thus have a large potential for implementation, is an innovative way to extend the shelf life, especially of raw meat, without a negative impact on its quality. This is of particular importance for food security (especially in the quantitative and qualitative aspects of food, but also in terms of food safety).

## 1. Introduction

The preservation of meat is a challenge for food producers not only because the composition of meat makes it a perishable material but also because meat is very sensitive to the loss of sensory properties during typical thermal processes [1]. In the thermal processing of meat, there are frequent changes in the structure of the food, a loss of consistency, and moreover, lipid oxidation, which is the main cause of rancid odors during storage [2].

Today, the growing demand for minimally processed foods that are both nutritionally and organoleptically satisfactory and free of microbiological hazards challenges research and development to identify alternative methods to reduce the level of bacterial contamination. The group of non-thermal technologies includes pulsed light (PL). It is a technology for the fast, mild, and residue-free surface decontamination of food and food contact materials in the processing environment [3]. This technology, discovered in the 1930s and whose first patent dates back to 1984 (Patent No. US4464336 A, 1984), is aimed at microbial inactivation to preserve food [4].

The term “pulsed light” has been known since 1980 and was first adopted by the US Food and Drug Administration (FDA) for food processing in 1996 [5]. PL is known by several synonyms, such as PL [6], pulsed white light (PWL) [7], pulsed UV-light [8], intense pulsed light (IPL) [9], high-intensity pulsed UV light (HIPL) [10], and high intensity broad spectrum pulse light [11]. Pulsed light technology is based on the use of very short light pulses in the range of 1 µs to 0, 1 s with a wide spectrum (from ultraviolet to near infrared, 100–1100 nm). High-power light pulses that operate for a short time are a powerful tool for inactivating microorganisms on food surfaces and packaging materials by combining both photochemical and photothermal mechanisms with no apparent effect on product properties [12,13]. This method is particularly useful for meat products that are cooked or dry cured and ready to eat [14]. Ready-to-eat products can pose a risk to the consumer due to post-processing, i.e., cutting, slicing, or packaging, during which pathogenic bacteria can reach the surface. In such foods, the most common pathogens present are *Listeria monocytogenes* and *Salmonella enterica* [15]. PL is an excellent method used in packaging disinfection and is also used to decontaminate tools in production facilities, preventing cross-contamination [16,17]. It also allows for the increase of various product characteristics, such as color, bioactive compound content, odor, and antioxidant activity [18,19].

The advantages of using pulsed light are primarily speed, low energy consumption, and environmental friendliness [4]. Working with pulsed light is also safe, as the process takes place in a chamber that has no direct contact with the environment. The PL technique can be applied alone or in combination with another method to maximize the effect [7,20,21].

The above indicates that PL processing, as a method with low energy consumption, can be environmentally friendly and, thus, have a large potential for implementation. It can be an innovative way to extend the shelf life, especially of raw meat, while remaining without a negative impact on its quality, which can be important for food security.

The minimal temperature effects of non-thermal technologies such as PL consequently pave the way for desirable, nutritious, organoleptically satisfactory, and at the same time minimally processed foods that are free from pathogens and food spoilage microorganisms [10,22,23,24].

The aim of this study was to investigate the effects of pulsed light application on the physicochemical, technological, and sensory properties, as well as the nutritional value and shelf life of cold-stored raw pig longissimus dorsi muscle. This would allow for demonstrating the usefulness of this method for extending the shelf life of raw meat without a negative impact on its quality characteristics, which will enable obtaining an innovative, environmentally friendly method of meat preservation thanks, among other things, to low energy consumption.

The use of a pulsating light stream will extend the durability of the longest back muscle in pigs. As a result, it will be possible to extend the shelf life of raw pork by min. 30% without any negative impact on its quality.

## 2. Materials and Methods

### 2.1. Samples Origin and Preparation

The research material consisted of samples of the longissimus dorsi muscle—(*m. longissimus dorsi*) obtained from pork half-carcasses from individual farmers associated in producer groups with contracts with a meat plant in the region of southeastern Poland. The fattening pigs were kept under the same environmental conditions and fed the same complete feeding mixtures. The animals had unlimited access to water. The study included meat from 40 fattening pigs (hybrids of the ♀ Polish Landrace × ♂ Duroc breeds), of which 50% were gilts and 50% were hogs. The carcass weight of slaughtered fatteners ranged from 110 to 120 kg. Animals after transport were kept in livestock stores for about 5 h. Slaughtering was carried out in accordance with the methodology of the meat industry. Before slaughter, the animals were subjected to electrical stunning. After 24 h of cold storage (+3 °C ± 0.5 °C), the longissimus dorsi muscle from the segment between the 4th and 5th thoracic vertebrae and the last lumbar and first sacral vertebrae was trimmed from the right half-carcasses. Each muscle was divided into six pieces across the muscle fibers. The size of the pieces was 50 × 50 × 50, three of which were control samples, and the rest were exposed to pulsed light (exposure to a pulsed light beam at 400 Hz for a period of 60 s with an energy dose of 600 mW and wavelengths of 660 and 405 nm). Both sides were exposed to PL. The distance between the light source and surface was 20 cm, and the area-related energy input of the PL was 6 J/cm^2^ within 1 min, which corresponds to the power density of 100 mW/cm^2^. Detailed laboratory tests of the meat were conducted 1, 7, and 10 days after slaughter. The meat was cold stored at +3 °C ± 0.5 °C. Starting from the 8th day (standard raw meat stored cold, without packaging, has a shelf life of about 7 days after slaughter), inspection tests were conducted daily to assess the freshness of the meat. Meat odor and surface appearance were evaluated. If a foul (ammoniacal) odor and/or slimy appearance on the surface were found on meat samples after fixation treatments, the meat was withdrawn from further cold storage and testing. After each period of cold storage, the following was performed on the control samples and after the application of pulsed light:

1. analysis of chemical composition (content: protein, fat, water, minerals, and salts);
2. measurements of pH, water activity, forced drip, thermal drip, oxidation-reduction potential, TBARS index, microbiological analysis-total microbial counts;
3. determination of color, browning index (BI), and heme pigment content;
4. determination of texture parameters and shear force;
5. sensory analysis (aroma: intensity and desirability, juiciness, tenderness, taste: intensity and desirability).

**Figure 1:**
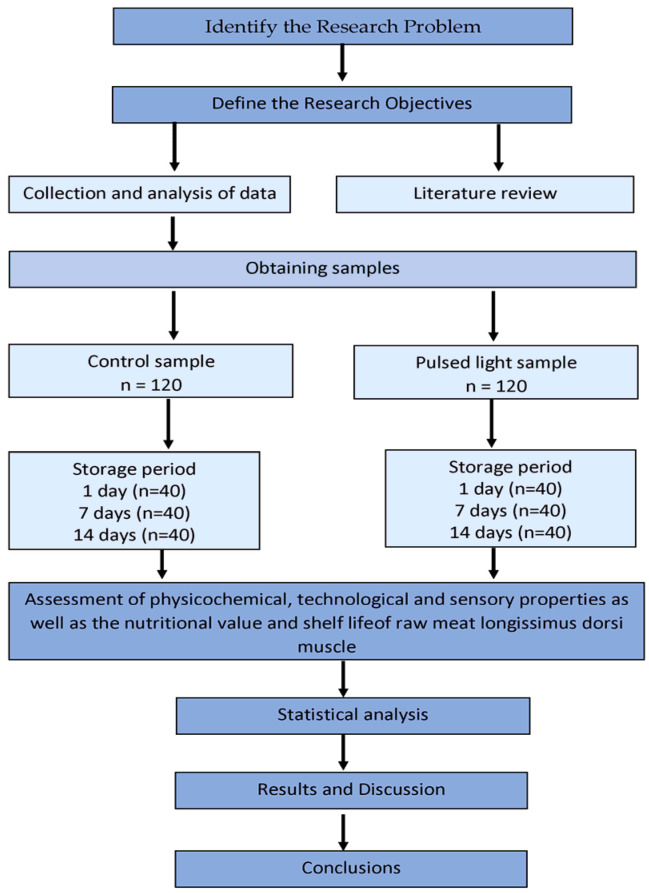
Schematic diagram of the experimentation.

Figure 1 shows schematic diagram of the experimentation.

### 2.2. Chemical Composition

In order to determine the chemical composition (water, fat, protein, minerals, and salt content), the samples were ground in a laboratory grinder (Zelmer, Rzeszów, Poland) using a mesh with a 4 mm hole diameter.

The water, protein, fat, salt, and mineral content was determined in accordance with the following norms: PN-ISO 1442: 2000 [25], PN-75/A-04018 [26], PN-ISO 1444: 2000 [27], PN-A-82112:1973 + Az 1:2002 [28], and PN-ISO 936: 2000 [29].

### 2.3. Physicochemical and Microbiological Properties

The active acidity (pH) of refrigerated meat was determined using an OSH 12-01 electrode and a CPC-411 pH meter (ELMETRON, Zabrze, Poland) with an accuracy of 0.01. The device was calibrated using buffers with pH values of 4.00 and 7.00.

Water activity (a_w_) is the ratio of the partial vapor pressure over the test sample to the partial vapor pressure over perfectly pure water. Water activity was measured on a LabMaster water activity measuring apparatus—aw (Novasina AG, Lachen, Switzerland). A 5 g sample was placed in a measuring vessel and closed with a lid. The lid was immediately removed from the sample container before being placed in the measuring chamber of the apparatus. The time from removing the cover to placing the sample in the apparatus and closing it again was 5 s. Measurements were performed at 20 °C. After the measurement was completed, the water activity value of the test sample was read from the instrument’s display.

The oxidation-reduction potential (EH, mV) was measured using an ERPt-13-type combination electrode and an ELMETRON CPC-505 waterproof pH/conductometer (Zabrze, Poland). Ten grams of the sample were homogenized in a T25 digital Ultra Turrax homogenizer (IKA Germany) for 1 min at a spindle speed of 15,000 rpm with 50 mL of deionized water. The redox potential of the obtained suspension was measured at 20 °C. The potential of the indicator electrode was related to the potential of an EODN reference semi-cell with an Ag/AgCl, 3 M KCl scheme used in the ERPt-13 electrode for a temperature range of 10–20 °C.

The total number of microorganisms was determined in accordance with PN-EN ISO 4833 [30]. This is a plate method that counts the number of microorganisms grown on PCA medium.

The determination of the TBARS index was based on the determination of a group of substances that form colored complexes with 2-thiobarbituric acid, such as, among others, malondialdehyde. The determination is based on the formation of colorful complexes of aldehydes present in fat with a solution of 2-thiobarbituric acid at high temperature. The intensity of the resulting coloration of the solution of the meat product with 2-thiobarbituric acid was measured spectrophotometrically at 532 nm. The reference solution was a control sample [31]. A 10 g sample was homogenized for one minute in a T25 digital Ultra Turrax homogenizer (IKA, Königswinter, Germany) at a spindle speed of 9500 rpm with 34.25 mL of 4% perchloric acid and 0.75 mL of an alcoholic solution of butylated hydroxytoluene. The homogenate was filtered through Whatman No. 1 filter paper. Then, 5 mL of the filtrate was taken into a test tube, and 5 mL of a 0.02 molar aqueous solution of 2-thiobarbituric acid was added, heated in a water bath for 60 min, and then cooled to room temperature. The measurement was made at a wavelength of 532 nm.

The amount of thermal drip was determined using the method of Janicki and Walczak [32]. 20 g of shredded meat, formed into a ball, was wrapped in gauze (a bandage), tied with wire, and placed in water at 85 °C for 10 min. After removal from the water, removal of the gauze, and cooling for 30 min at 4 °C, the meat samples were weighed again. Thermal drip was calculated from the difference in weights before treatment and after cooling according to the formula:

(1) $$\mathrm{Td}\;(\%)=\frac{\mathrm{WI}-\mathrm{WII}}{\mathrm{WI}}\;\times \;100\%$$

where: Td—amount of thermal drip (%), WI—weight of sample before heat treatment (g), WII—weight of sample after heat treatment and after cooling (g).

The meat forced drip was determined using the method of Grau and Hamm [33] by placing the ground sample (about 300 mg) on Whatman blotting paper No. 1. The blotting paper and the sample were inserted between two glass plates and subjected to 2 kg of pressure for a period of 5 min. After the presumed pressing time, the boundary of the area occupied by the meat sample and the drip of meat juice were outlined on the blotting paper, which was then planimetered. The measure of the amount of forced meat juice drip was the difference between the two surfaces, which was the result of interpreting the water absorption (cm^2^) (higher value—lower water absorption of meat).

### 2.4. Color Measurement and Heme Pigment Content

Instrumental color measurement in the CIE L*a*b* system was performed over the entire surface of the muscle sample using a HunterLab UltraScan PRO electronic colorimeter (HunterLab, Reston, VA, USA) (light source D65, measuring head opening 20 mm, calibration with the white standard: L*—99.18, a*—0.07, b*—0.05). In this system, L* stands for brightness, which is a spatial vector, while a* and b* are trichromaticity coordinates, where positive values of a* correspond to red, negative values to green, positive b* to yellow, and negative b* to blue.

However, the browning index (BI) was calculated using Equation, and represents the purity of brown color in pigs’ Longissimus dorsi muscle. Changes in this index (BI) can be linked to changes in the chemical and organoleptic properties of food [34].

$$\mathrm{BI}=\frac{a^{*}+1.75\cdot L^{*}}{5.645\cdot L^{*}+a^{*}-3.012\cdot b^{*}}-0.31$$

On the basis of the experimentally obtained parameters, the total color difference (∆E) between the control sample and the PL-samples was calculated according to the following equation:

$$\Delta E=\sqrt{{\left(\Delta L\right)}^{2}+{\left(\Delta a\right)}^{2}+{\left(\Delta b\right)}^{2}\;}$$

Depending on the (∆E values, the color difference can be classified as: not noticeable (0–0.5), slightly noticeable (0.5–1.5), noticeable (1.5–3.0), well visible (3.0–6.0), and highly visible (6.0–12.0) [35].

The percentage of heme dyes was determined by extraction with cold phosphate buffer (0.04 M, pH 6.8) and clarification by centrifugation (at +4 °C) for 60 min at 15,000 15 rpm. After clarification, absorbance was measured at wavelengths of 525 nm, 545 nm, 565 nm, and 572 nm. The reference solution for the measurement was phosphate buffer [36].

### 2.5. Texture Measurement

In order to determine the texture parameters of the tested meat, cube-shaped samples with 20 mm sides were cut from each batch of meat. Instrumentally, the texture parameters of the tested meat samples were determined using Texture Profile Analysis (TPA) performed with a Texture Analyser—CT3—25 (Brookfield, WI, USA), with a 38.1 mm diameter and 20 mm long cylindrical attachment. A 2-fold compression test was performed on the samples at 50% of their height. The speed of the cylinder during the test was 2 mm/s, while the interval between pressures was 2 s. The following texture parameters were determined using Texture Pro CT v. 1.9 software (Brookfield, WI, USA): hardness 1, hardness 2, rigidity up to 5 mm, rigidity up to 8 mm, adhesiveness, resilience, cohesiveness, springiness, gumminess, and chewiness. During serial measurements, all texture parameters were counted automatically.

The shear force of raw meat was determined using a Warner-Bratzler TA.XT plus texture-meter (Stable Micro Systems Ltd., Surrey, UK). Cylindrical raw meat samples, cut with a 1.0-cm-diameter corkboard (along the muscle fibers), were cut with a Warner-Bratzler blade with a triangular notch, and the value of the force required to cut them (N/cm^2^) was recorded. The average value from three consecutive repetitions was taken as the final measurement of each sample.

### 2.6. Sensory Properties

The sensory properties of pork meat were evaluated according to the methodology provided by Baryłko-Pikielna and Matuszewska [37]. A 100 g meat sample was heat-treated in a water bath by Funke Gerber WB436-D (Berlin, Germany) at 100 °C until an internal temperature of 80 °C ± 2 °C was reached. The temperature was measured inside using a thermometer (Sous Vide Thermapen, MERA, Warsaw, Poland) equipped with a needle probe. For sensory evaluation, the heat-treated samples were cooled to 20 °C ± 2 °C and cut into 1.5 cm thick slices across the muscle fibers. All samples to be evaluated were in covered plastic dishes, marked with individual digital codes. Samples for sensory evaluation were taken in random order. Evaluators conducted sensory evaluations in triplicate. Sensory quality evaluation of meat was carried out by a permanent, 6-member laboratory team, which checked for sensitivity and sensory performance in accordance with ISO 8586-2 [38] and ISO 8587 [39]. The evaluation team consisted of 6 people (50% male/female, ages 30 to 56). The evaluators were experienced in evaluating meat and processed meat products. A 5-point partial quality sensory evaluation was used, assessing the following quality indicators: intensity of aroma (1 = very negative, very weakly perceptible, 5 = very strong), intensity of taste (1 = very negative, very weakly perceptible, 5 = very desirable), desirability of aroma (1 = undesirable, 5 = highly desirable), desirability of taste (1 = undesirable, 5 = highly desirable), juiciness (1 = very dry, 5 = very juicy), and tenderness (1 = very tough, 5 = very tender). The evaluation of the sensory properties of meat was carried out in a sensory laboratory that met all the requirements of the relevant standard [40]. Between each examination of the meat samples, the evaluators used a 30-s pause to rinse their mouths with mineral water.

### 2.7. Statistical Analysis

All determinations and sensory evaluations were carried out in triplicate. The results obtained were grouped and subjected to statistical calculations. The tables show the means and standard errors. Statistically significant differences between the mean values were determined using the NIR (least significant difference) test with a significance level of *p* < 0.05. Calculations were performed using Statistica ver. 13 software.

## 3. Results and Discussion

The share and proportions of basic chemical components determine not only the nutritional value of meat but also its consumer appeal. Table 1 shows the results of the chemical composition analysis (content of protein, water, fat, minerals, and salts) of the longissimus dorsi muscle after cold storage and the application of pulsed light. These data show that the content of the analyzed chemical components did not change significantly after the application of a pulsed light beam. There are no data in the literature on current studies of the effects of pulsed light beams on the chemical composition of the longissimus dorsi muscle.

The physicochemical and microbiological properties of longissimus dorsi muscle are shown in Table 2. Both the meat pH results and the thermal drip and forced drip testify to the freshness and good hydration properties of the meat used in the study. The pH values of the longissimus dorsi muscle obtained in the study showed no significant effect of PL on acidity on the first, seventh, and tenth days of cold storage. It was observed that the applied PL caused a slight reduction in pH values during the entire storage period compared to the control sample. In addition, the study showed that PL had no statistically significant effect on forced drip, thermal drip, or total microbial count. Data in Table 2 indicates that there was a decrease in the total microbial count after pulsed light application compared to the control sample during the different periods of cold storage, but the changes were not statistically significant. Inactivation of microorganisms is a non-selective process in which the most important mechanism is the photochemical effect. The degree of reduction is determined by the type of meat, its chemical composition, and the type and physiological state of microorganisms [41,42]. The effect of the pulsating light stream, according to the literature [41,42,43], is damage to cell membranes, which is manifested by a decrease in the total number of microorganisms. Similarly, count reductions of selected pathogens by approximately 1 log were previously found by Hierro et al. [18] in the cases of PL treated tuna and beef carpaccio, but distinctively higher fluences of up to 8.4 and 11.9 J/cm^2^ were applied. The same research group reported maximum log reductions for *L. monocytogenes* on packaged sliced cooked ham and bologna of 1.11 cfu/cm^2^ and 1.78 cfu/cm^2^ after treatments at 8.4 J/cm^2^, respectively [14]. Kramer et al. [44] show that PL treatment allows reductions of *L. innocua* by about 1 log on the surface of sliced boiled ham and chicken cold cuts, while 3–4 logs can be achieved on frankfurter sausages. Furthermore, Ganan et al. [45] found reductions of *L. monocytogenes* and *S. Typhimurium* on the surface of ready-to-eat dry cured meat products (salchichón and loin) by 1.5–1.8 log cfu/cm^2^ at a fluence of 11.9 J/cm^2^.

Water activity, in turn, determines the course of biological processes in meat, including microbial growth [46]. Analyzing the results obtained, a decrease (*p* < 0.05) in water activity values was observed after PL was applied to LD muscle on the tenth day of cold storage. On the first and seventh days of this raw material storage, water activity in both control and PL-treated samples was at similar levels, ranging from 0.975 to 0.980.

An important factor affecting meat color is redox potential, which determines the redox status of iron centrally located in the porphyrin ring of the myoglobin molecule [47]. The oxidation-reduction potential (ORP) value of meat depends on the concentration of oxidants and reductants present in the meat. The more oxidants there are in the meat, the higher the ORP values obtained, and when the amount of oxidants decreases and the concentration of reductants increases, the ORP values are lower [48]. From the data in Table 2, it can be seen that after the application of PL, the oxidation-reduction potential values of meat were lower after the seventh and tenth days of cold storage compared to the control sample. However, statistically significant differences were observed only on the seventh day of refrigerated storage of this raw material.

Based on the tests performed (Table 2), there was a decrease (*p* < 0.05) in the values of the TBARS index of LD muscle after 1 and 7 days of cold storage of this raw material. On the other hand, after 10 days of cold storage, the values of this index were at a similar level. Lipids are considered to be one of the most chemically unstable food ingredients because they are easily oxidized and auto oxidized [49]. The occurrence of lipid oxidation is highly undesirable as it leads to the formation of toxic components, the loss of nutritional properties, the development of rancidity, and an off flavor. The susceptibility of muscle tissues to lipid oxidation depends on the endogenous characteristics of the tissue, including its fat content, fatty acid profile, and tissue oxidative potential [50]. In the study, a decrease in TBARS values was observed on the 1st and 7th days of refrigerated storage, which reflects the very low oxidation of lipids. The use of a short pulsed light beam on meat could most likely reduce oxidative changes in lipids. When applied in pulsed form, the short duration of the pulse limits oxidative changes in lipids due to the short half-life of the p-bonds, which prevents efficient coupling with oxygen, which could explain the low 2-thiobarbituric acid-reactive substance levels observed in PL treated meat and meat products [51]. Tarlagdis et al. [52] reported a threshold range of 0.5–1.0 μg MDA/g pork for the sensory detection of rancidity as determined by trained panelists. In a study conducted by Koch et al. [53], it was shown that in pork skin, the MDA concentration was significantly higher (*p* < 0.05) at fluences of 0.84, 9.66, and 14.56 J/cm^2^ in comparison to the control and the other fluences applied, but clearly remained below the threshold concentration of 0.5 μg/g. In loin, there is no statistically significant difference between the control and treated samples. These results are in agreement with the work of Rajkovic et al. [54], who did not find any significant differences in the concentration of MDA between the control (0.33 mg MDA/kg), 3 J/cm^2^ (0.31 mg MDA/kg), and 15 J/cm^2^ treated salami samples (0.49 mg MDA/kg). Nicorescu et al. [55], who studied the effect of pulsed light on the organoleptic properties and shelf-life extension of pork and salmon, show that malondialdehyde (MDA) content substantially increased in RS (raw salmon) and RP (roasted pork) samples submitted to 30 J cm^−2^, i.e., by 39.3% and 25.5%, respectively.

The color of meat is one of the basic distinguishing features of its technological and culinary quality and is one of the most important differentiating factors in the consumer assessment of this raw material [56,57,58,59]. The muscle pigment responsible for the color sensation is myoglobin, which is oxidized to oxymyoglobin or oxidized to metmyoglobin. According to many authors [60,61,62], the color of meat is influenced by two main factors: its acidity and fat content, which cause the so-called marbling of meat. The results of the analysis on the application of pulsed light beam on the L*, a* and b* color parameters, total color difference (∆E), browning index (BI) and heme pigment content of the longissimus dorsi muscle are shown in Table 3. A statistically significant (*p* < 0.05) effect of PL on the increase in color brightness was found, but only for meat on the 10th day of cold storage. The pulsed light beam applied to the meat had no significant effect on the variation of color parameters a* and b* or the browning index. The total color difference after 1 day of storage was barely noticeable (∆E = 1.43). While the longer the storage time, the ∆E was noticeable or even clearly visible. In the case of heme pigments, there was also no statistically significant effect of PL on changes in the content of individual forms. However, it should be noted that, especially after 10 days of cold storage, the values of the oxidized form (METMB) decreased while the amount of oxymyoglobin (MBO) increased. The increase in color brightness in meat after 10 days of refrigerated storage was most likely associated with a higher proportion of oxidized myoglobin-oxymyoglobin (MBO) and a lower proportion of oxidized myoglobin-methylmyoglobin (METMB). Similar reports were made by Ganan et al. [45], who studied the efficacy of PL for the inactivation of *L. monocytogenes* and *S. enterica* on two RTE dry cured meat products (salchichón and loin) in view of a future application of this technology to improve their microbiological safety and shelf-life. In salchichón, lightness (L*) was significantly higher (*p* < 0.05) in samples treated with 11.9 J/cm^2^ right after the treatment, but these differences disappeared during storage due to a slight increase of L* in the rest of the samples. The red (a*) and yellow (b*) values of salchichón were not significantly affected by the treatment. Various studies on the use of other non-thermal technologies to dry cure meats, such as irradiation or high-pressure treatment, were also reported in a* as L* increased [63,64,65,66]. In addition, in some of these studies, color differences tended to disappear during storage.

The lightness of color L* determines the total amount of light reflected from the surface of the slice of meat [67]. It depends to a large extent on the water holding capacity, the structure of the meat tissue, its acidity (pH), and the depth of penetration of the light beam [68,69]. A study by Koch et al. [53] showed that the L* value of the PL treated loin was not affected by any of the selected parameter combinations. Loin samples became significantly less red when treated at 9.66, 12.81, and 19.11 J/cm^2^. ΔE*ab did not exceed the value of 3 on either of the tested fluences.

A number of other research groups have made the same observations regarding redness. Almost all tested meat types showed a decrease in a* values except for chicken and fish [14,55,70,71,72], which might be attributable to the small amounts of chromophores in chicken and fish.

Myoglobin is the main pigment found in the muscle tissues, and its content depends on the source and type of meat. On the other hand, the concentration of myoglobin in the muscle cells of beef, pork, and chicken breast is under 0.05%, 0.1–0.3%, and 1.5–2.0%, respectively [55]. The data in Table 3 show that after PL application, myoglobin values are lower compared to the control sample throughout the storage period. Genot [73] has shown that the acceleration of myoglobin oxidation in meat leads to important color changes. Therefore, the difference in the color changes observed between the pork, beef, and chicken could be explained by the co-oxidation of liposoluble or water-soluble pigments induced by PL treatment.

Texture is arguably the most important quality factor associated with consumer satisfaction when eating meat products [74]. Texture profile analysis takes into account the multi-parameter properties of the product and the classification of mechanical texture parameters. The main texture parameters include: hardness, cohesiveness, springiness, resilience, adhesiveness, and gumminess. Hardness is the force required to achieve the desired deformation; springiness is the rate of return from the deformed state to the initial state; chewiness is the energy required to break down solid products; and gumminess is the energy required to plasticize a piece of meat to a swallowable state [75]. Secondary parameters of texture include chewiness, that is, the energy required to grind (chew) the product. This parameter is related to hardness, cohesiveness, and springiness [76]. Table 4 shows the results of the texture parameters of cold the stored longissimus dorsi muscle after the application of pulsed light. The study showed a statistically significant (*p* < 0.05) effect of PL on the variation of such texture parameters as hardness 1 and 2, rigidity, and chewiness, but only for muscles that had been cold stored for 10 days. For the other texture parameters, i.e., springiness, cohesiveness, adhesiveness, resilience, and gumminess, no statistically significant differences were shown. Lower values of some parameters of the texture of meat subjected to a pulsed light stream after 10 days of refrigerated storage may be most likely caused by changes in the structure of meat proteins and a faster rate of their transformation.

In the instrumental evaluation of meat texture, the most commonly used parameter, which is interdependent with tenderness, is the value of the maximum shear force obtained from the shear test. Meat tenderness is considered by consumers to be one of the most important characteristics of meat quality. The value of shear force is influenced by a number of live and post-mortem factors, such as species, breed, age, sex, individual characteristics, and animal housing systems [77]. Bratcher et al. [78] and White et al. [79] observed a significant effect of slaughtering and carcass-cooling conditions, as well as the storage and maturation of meat, on its mechanical resistance. Table 4 shows the results of the study on changes in the shear force values of the longissimus dorsi muscle subjected to PL during cold storage. These data show that after the application of pulsed light, the shear force values were lower (*p* < 0.05) compared to the control sample after the first day of cold storage. The differences in terms of this trait were statistically insignificant during the remaining periods of cold storage of LD muscle. The literature lacks data on current studies of the effect of a pulsed light beam on the texture of the longissimus dorsi muscle.

**Figure 2:**
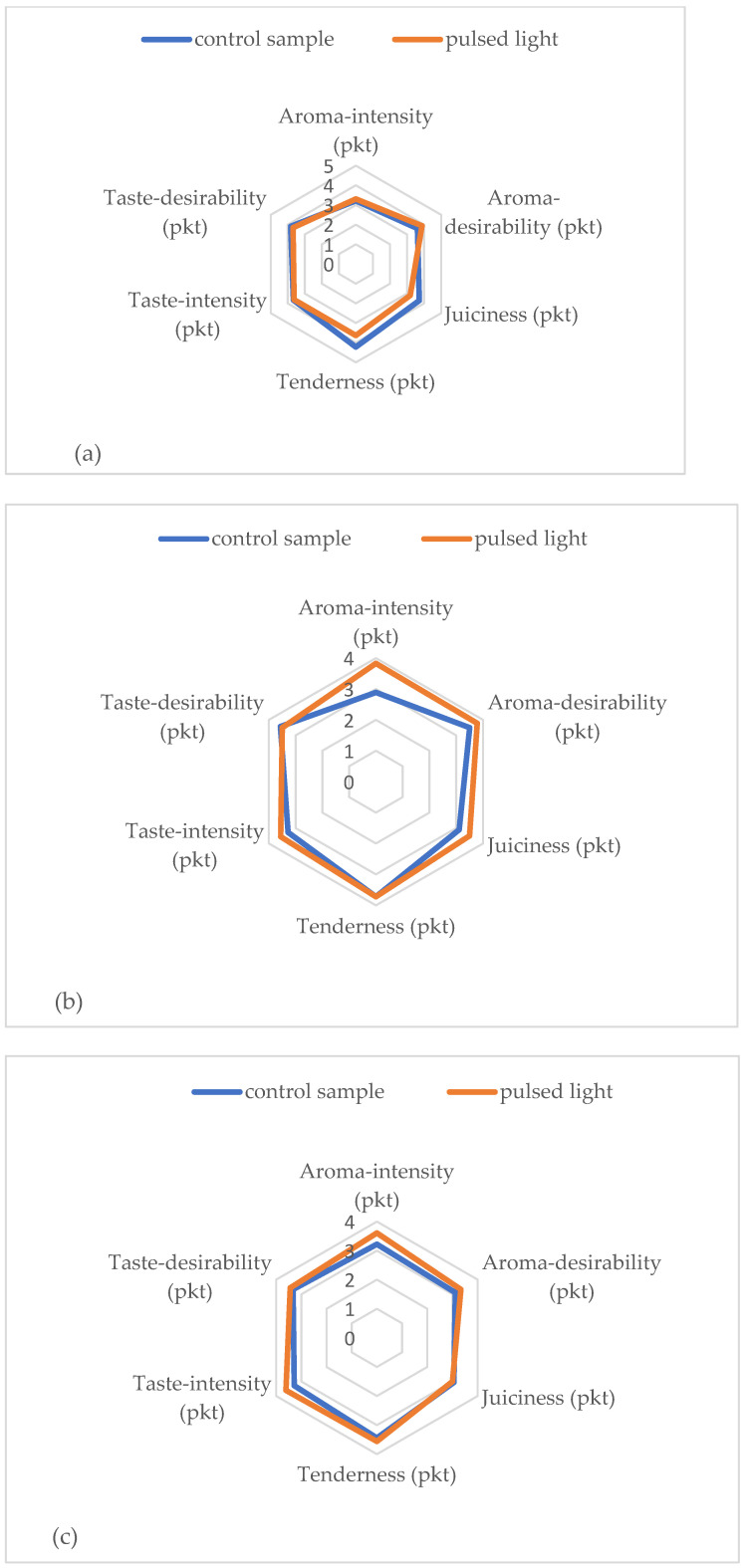
Sensory analysis of the longissimus dorsi muscle after cold storage and pulsed light application (**a**) 1 day cold storage, (**b**) 7 days cold storage, (**c**) 10 days cold storage.

The sensory analysis is defined by Barilko-Pikielna [80] as the measurement and evaluation of the properties (qualitative characteristics) of a product by means of one or more senses used as measuring apparatus, with appropriate evaluation conditions and requirements for those conducting the evaluation, as well as methods adapted to the tasks of evaluation. Table 5 and Figure 2 show the results of testing the sensory properties of the longissimus dorsi muscle after cold storage and the application of pulsed light. These data show that the application of PL did not have a statistically significant effect on the variation in the perception of selected sensory characteristics of meat. However, it should be noted that, in terms of intensity and desirability of aroma, PL-treated meat scored slightly higher. By analyzing the results of the odor intensity assessment of the longissimus dorsi muscle, a beneficial effect of the pulsating light stream was observed. Aroma-intensity scores in the PL-treated samples were slightly higher compared to the control samples. On the 7th day of storage, the aroma-intensity of the meat samples subjected to PL was assessed at 3.83 points, and in the control sample, it was 2.89 points (differences were statistically insignificant). A similar relationship was found in the case of point evaluation of aroma-desirability in the tested samples. On days 1 and 7 of refrigerated storage, the taste desirability score in the samples exposed to the pulsating light stream was lower. However, after 10 days of storage, the point evaluation of taste desirability in meat treated with PL was slightly higher and amounted to 3.44 points compared to the control sample—3.33 points (*p* > 0.05). The research conducted by Tomasevic and Rajkovic [71] showed that PL had a significant impact only on the smell of the tested samples of meat, poultry, and venison. Ganan et al. [45] showed in their research that, in addition to confirming the effectiveness of the method in reducing microorganisms on the surface of meat products, dried RTE also showed that pulsed light does not significantly affect the change in sensory properties of preserved products. Tomasevic [70] showed that the sensory quality of 1-pulse treated fermented sausage was not significantly different to the untreated fermented sausage. A study by Ozer and Demirci [81] also noticed that a PL treatment of 5.6 J/cm^2^ did not affect the quality of salmon fillets.

Regarding the impact on sensory properties, the study carried out by Koch et al. [53] indicates that the most intense PL treatments (4.96 and 12.81 J/cm^2^) are associated with unpleasant, ozoneous, pungent, ammoniacal, and off-odor perceptions in pork skin and loin. Conversely, the samples subjected to 0.52 J/cm^2^ were perceived as “less porky” and “slightly chemical,” which support the indication that excessive PL treatment reduced the quality of food. Moreover, Hierro et al. [18] assessed the feasibility of PL treatments (0.7, 2.1, 4.2, 8.4, and 11.9 J/cm^2^) to enhance the safety of beef and tuna carpaccio. However, PL treatments at high doses (8.4 and 11.9 J/cm^2^) resulted in color variation (lower redness and yellowness than the untreated sample) and a negative impact on sensorial quality (lower scores for color and odor in comparison to the untreated sample).

## 4. Conclusions

The use of a pulsed light beam had a beneficial effect on some quality characteristics of the longissimus dorsi muscle. For example, the treatment caused a decrease (but statistically insignificant differences) in the total number of microorganisms in the meat compared to the control sample during the various periods of cold storage. In addition, water activity values were lower (*p* < 0.05) on the tenth day of storage after the PL treatment. There was also a decrease in meat oxidation-reduction potential values after the seventh and tenth days of cold storage and lower TBARS values after the first and seventh days of cold storage, indicating improved anti-oxidation properties in LD muscle during the aforementioned periods of cold storage (*p* < 0.05). Accordingly, the treatment can extend the shelf life of raw pork by about 30%. The application of PL also resulted in an increase (*p* < 0.05) in the brightness of the meat color after the tenth day of cold storage. This was most likely related to a higher proportion of the oxidized form of myoglobin-oxymyoglobin (MBO) and a lower proportion of the oxidized form of myoglobin-methyl myoglobin (METMB) in meat after the application of this treatment after the 10th day of cold storage. However, verifying this requires further, more specialized studies.

## Figures and Tables

**Table 1:** Chemical composition of the longissimus dorsi muscle after cold storage and pulsed light application.

Specification	Cold Storage Period (Days)					
	1		7		10	
	K	S	K	S	K	S
Protein (%)	20.85 ± 0.31	20.75 ± 0.31	20.25 ± 0.31	20.37 ± 0.31	20.43 ± 0.31	20.32 ± 0.31
Fat (%)	7.05 ± 1.46	7.16 ± 1.12	7.25 ± 1.21	7.19 ± 1.45	7.34 ± 1.32	7.26 ± 1.13
Water (%)	71.01 ± 1.28	71.23 ± 1.32	71.43 ± 1.45	71.35 ± 1.67	70.95 ± 1.63	70.98 ± 1.84
Minerals (%)	1.49 ± 0.08	1.50 ± 0.30	1.46 ± 0.16	1.54 ± 0.40	1.52 ± 0.16	1.58 ± 0.13
Salts (%)	0.57 ± 0.02	0.57 ± 0.02	0.61 ± 0.07	0.65 ± 0.15	0.49 ± 0.06	0.48 ± 0.03

Notes: K—control sample; S—pulsed light.

**Table 2:** Physicochemical and microbiological properties of the longissimus dorsi muscle after cold storage and pulsed light application.

Specification	Cold Storage Period (Days)					
	1		7		10	
	K	S	K	S	K	S
pH	5.55 ± 0.05	5.53 ± 0.07	5.55 ± 0.02	5.54 ± 0.04	5.61 ± 0.12	5.58 ± 0.07
Water activity	0.976 ^ab^ ± 0.003	0.980 ^ab^ ± 0.004	0.975 ^ab^ ± 0.003	0.977 ^ab^ ± 0.001	0.976 ^a^ ± 0.004	0.957 ^b^ ± 0.010
Thermal drip (%)	21.10 ± 1.17	21.64 ± 2.28	22.88 ± 2.77	20.63 ± 2.11	19.47 ± 1.76	18.51 ± 1.18
Forced drip (cm^2^)	2.83 ± 0.70	2.66 ± 0.41	2.11 ± 0.78	3.73 ± 0.86	3.57 ± 0.14	2.79 ± 0.05
TBARS index (mg MDA/kg)	0.46 ^a^ ± 0.09	0.35 ^b^ ± 0.05	0.61 ^a^ ± 0.11	0.52 ^b^ ± 0.03	0.56 ^ab^ ± 0.05	0.57 ^ab^ ± 0.10
Oxidation-reduction potential (mV)	358.50 ^ab^ ± 5.02	365.57 ^ab^ ± 5.62	443.67 ^a^ ± 14.10	414.67 ^b^ ± 1.67	414.83 ^ab^ ± 12.03	404.30 ^ab^ ± 6.30
Total numer of microorganisms (CFU/g)	6.82 × 10^2^ ± 5.57 × 10^2^	5.30 × 10^2^ ± 1.94 × 10^2^	7.50 × 10^3^ ± 4.54 × 10^2^	3.63 × 10^3^ ± 1.31 × 10^2^	3.10 × 10^6^ ± 2.43 × 10^5^	1.59 × 10^6^ ± 1.33 × 10^5^

Notes: K—control sample; S—pulsed light; ^a,b^—Differences in pairs statistically significant at the level *p* < 0.05, ^ab^—no letters or the same letters next to the means indicate no statistically significant differences.

**Table 3:** Color parameters, browning index (BI), total color difference (∆E) and heme pigment content of longissimus dorsi muscle after cold storage and pulsed light application.

Specification	Cold Storage Period (Days)					
	1		7		10	
	K	S	K	S	K	S
L*	53.87 ^ab^ ± 3.67	52.90 ^ab^ ± 2.60	54.86 ^ab^ ± 2.34	57.14 ^ab^ ± 4.12	53.18 ^a^ ± 5.91	58.51 ^b^ ± 3.70
a*	11.42 ± 0.76	10.84 ± 0.48	13.06 ± 1.70	12.60 ± 1.52	11.67 ± 1.49	11.83 ± 1.13
b*	6.84 ± 0.77	7.71 ± 1.86	8.48 ± 1.28	8.30 ± 1.02	8.12 ± 1.12	8.65 ± 1.08
Browning index (BI)	28.46 ± 2.51	30.17 ± 3.05	33.51 ± 2.34	31.19 ± 2.89	32.00 ± 2.71	30.23 ± 3.12
∆E	1.43		2.33		5.36	
MB (%)	60.05 ± 6.63	45.80 ± 10.93	51.29 ± 6.21	48.90 ± 8.43	43.45 ± 12.65	42.98 ± 21.37
METMB (%)	17.19 ± 7.25	30.00 ± 11.65	28.63 ± 9.38	27.44 ± 6.97	30.82 ± 8.17	21.03 ± 8.70
MBO (%)	22.76 ± 2.68	24.20 ± 9.13	20.08 ± 5.19	23.66 ± 7.07	25.73 ± 10.59	35.99 ± 18.33
OZB (mg/kg)	6.84 ± 0.87	7.21 ± 1.14	7.01 ± 1.46	7.32 ± 0.91	7.09 ± 1.32	7.24 ± 0.96

Notes: K—control sample; S—pulsed light; ^a,b^—Differences in pairs statistically significant at the level *p* < 0.05, ^ab^—no letters or the same letters next to the means indicate no statistically significant differences.

**Table 4:** Texture parameters and shear force of the longissimus dorsi muscle after cold storage and pulsed light application.

Specification	Cold Storage Period (Days)					
	1		7		10	
	K	S	K	S	K	S
Hardness 1 (N)	91.91 ^ab^ ± 6.03	140.02 ^ab^ ± 5.21	156.34 ^ab^ ± 4.80	131.34 ^ab^ ± 3.76	132.35 ^a^ ± 8.82	47.23 ^b^ ± 4.89
Hardness 2 (N)	44.53 ^a^ ± 3.02	80.63 ^b^ ± 4.79	105.40 ^ab^ ± 8.58	83.65 ^ab^ ± 3.45	83.44 ^a^ ± 6.00	35.19 ^b^ ± 6.55
Rigidity 5 (N)	28.15 ± 2.02	43.10 ± 9.36	28.96 ± 1.46	26.51 ± 1.24	9.94 ± 1.98	5.30 ± 1.08
Rigidity 8 (N)	68.06 ^ab^ ± 5.47	110.13 ^ab^ ± 6.72	104.26 ^ab^ ± 4.28	78.40 ^ab^ ± 3.43	67.53 ^a^ ± 4.65	20.95 ^b^ ± 1.76
Adhesiveness (mJ)	1.33 ± 0.08	2.01 ± 0.01	2.39 ± 0.06	1.53 ± 0.02	1.86 ± 0.09	2.14 ± 0.03
Cohesiveness	0.19 ± 0.05	0.27 ± 0.06	0.19 ± 0.08	0.26 ± 0.03	0.26 ± 0.08	0.23 ± 0.06
Springiness(mm)	2.41 ± 0.58	2.92 ± 0.43	3.08 ± 0.37	3.94 ± 0.32	4.03 ± 0.71	3.60 ± 0.59
Resilience	0.14 ± 0.01	0.11 ± 0.02	0.15 ± 0.05	0.13 ± 0.06	0.21 ± 0.08	0.24 ± 0.03
Gumminess (N)	11.85 ± 1.10	15.92 ± 1.61	29.62 ± 2.46	35.94 ± 3.43	34.44 ± 2.78	15.08 ± 1.17
Chewiness (mJ)	30.80 ^ab^ ± 2.60	48.01 ^ab^ ± 2.81	91.98 ^ab^ ± 3.92	142.36 ^ab^ ± 2.06	144.72 ^a^ ± 5.71	39.47 ^b^ ± 5.74
Shear force (N/cm^2^)	102.10 ^a^ ± 20.71	69.41 ^b^ ± 19.77	81.40 ^ab^ ± 20.42	70.06 ^ab^ ± 9.52	64.07 ^ab^ ± 7.13	68.43 ^ab^ ± 2.30

Notes: K—control sample; S—pulsed light; ^a,b^—Differences in pairs statistically significant at the level *p* < 0.05, ^ab^—no letters or the same letters next to the means indicate no statistically significant differences.

**Table 5:** Sensory properties of the longissimus dorsi muscle after cold storage and pulsed light application.

Specification	Cold Storage Period (Days)					
	1		7		10	
	K	S	K	S	K	S
Aroma-intensity (pkt)	3.22 ± 0.71	3.31 ± 0.88	2.89 ± 0.70	3.83 ± 0.43	3.22 ± 0.62	3.61 ± 0.60
Aroma-desirability (pkt)	3.61 ± 0.49	3.88 ± 0.69	3.50 ± 0.71	3.78 ± 0.51	3.11 ± 0.55	3.33 ± 0.66
Juiciness (pkt)	3.72 ± 0.06	3.19 ± 0.70	3.11 ± 0.36	3.50 ± 0.66	3.06 ± 0.53	3.00 ± 0.61
Tenderness (pkt)	4.22 ± 0.71	3.63 ± 0.83	3.72 ± 0.51	3.72 ± 0.79	3.44 ± 0.85	3.56 ± 0.63
Taste-intensity (pkt)	3.67 ± 0.43	3.63 ± 0.44	3.28 ± 0.87	3.56 ± 0.73	3.28 ± 0.36	3.61 ± 0.42
Taste-desirability (pkt)	3.83 ± 0.35	3.69 ± 0.46	3.57 ± 0.95	3.50 ± 0.56	3.33 ± 0.50	3.44 ± 0.58

Notes: K—control sample; S—pulsed light.

## Data Availability

The authors declare that data or models are not deposited in an official repository.
